# Analyzing the labor market and salary determinants for big data talent based on job advertisements in China

**DOI:** 10.1371/journal.pone.0317189

**Published:** 2025-02-04

**Authors:** Yingjie Lu, Hong Tuo, Haoyi Fan, Haiying Yuan

**Affiliations:** 1 School of Economics and Management, Beijing University of Chemical Technology, Beijing, China; 2 School of Business Administration, Shanghai Lixin University of Accounting and Finance, Shanghai, China; IUB: The Islamia University of Bahawalpur Pakistan, PAKISTAN

## Abstract

The demand for big data talent is rapidly increasing with the growth of the big data industry. However, there has been limited research on what employers seek in recruiting big data talent. This paper aims to apply labor market segmentation theories to the big data labor market and develop a theoretical framework to analyze the distribution of big data talent in different labor market segments. Furthermore, we develop a salary determination model to explain wage differentials. An empirical analysis is conducted using online job advertisements from a Chinese recruitment website to investigate the labor market for big data talent in China. Our findings show that there are significant differences in the demand for big data talent across different types of cities and industries. Different types of enterprises have different requirements for individual characteristics and offer various levels of big data job positions. Furthermore, our results reveal that individual, job-related and organizational characteristics are all significant predictors of salaries. These findings can provide particularly useful insights for organizations and managers in the big data industry.

## Introduction

With the rapid advancement of information technology, society has entered the era of big data. According to research from McKinsey, a leading global consulting firm, big data will become a key basis for competition, underpinning new waves of productivity growth, innovation, and consumer surplus [1]. Increasing numbers of companies are becoming aware of the potential benefits of big data and are leveraging big data tools and technologies to support more effective and efficient decision making and to facilitate product and service innovation [2, 3].

In recent years, the demand for big data talent has increased dramatically, which has led to the supply of big data talent lagging far behind market demand. According to an article in Forbes Magazine [4], jobs related to big data have become one of the fastest growing segments in the overall job market. Another survey from the website MarketWatch.com showed that big data ranked at the top of the “Best Jobs in America” list in 2017. While there is rapidly growing demand for big data talent, the supply of talent is seriously insufficient [5]. A report from McKinsey noted that the gap between the demand and supply of big data talent in the United States is estimated to be larger than 50 percent [6]. The issue seems to be even more serious in China than in many other countries in the world [7]. With the rapid development of the IT industry in China, the application of big data-related technology in various fields, such as finance, commerce, entertainment, and healthcare, has received extensive attention [8]. However, there are now fewer than 300,000 people who engage in big data-related work. This serious shortage of big data talent and technical personnel is becoming an urgent problem [9].

In recent years, many scholars have become aware of this serious shortage of big data talent and have proposed valuable suggestions to increase the supply of such talent, e.g., offering more courses on big data to college students and developing optimized training programs for employees working with big data [10]. Despite these efforts, however, the mismatch between the supply of trained talent and the demands from enterprises remains a significant challenge. This imbalance is not merely a result of insufficient educational and training initiatives, but also stems from a lack of clarity regarding employers’ specific requirements for big data professionals [11, 12]. Especially in China, where the big data industry is experiencing rapid growth with significant technological advancements, the existing curriculum often fails to meet market demand [13]. Understanding the attributes and qualifications that employers prioritize in recruiting big data talent is essential for developing education and training strategies that are aligned with industry demands. Otherwise, educational efforts may diverge from actual labor market demands, resulting in a long-term gap between talents’ skills and employers’ expectations.

To fill this research gap, this study focuses on the market demand for big data talent in China and, by investigating the big data job market, attempts to understand what employers are looking for when recruiting big data talent.

Because big data is widely used in various industries with different needs for big data knowledge and skills [14, 15], it is difficult to gain a comprehensive and accurate understanding of the big data job market through traditional questionnaires or interviews with recruiters. Therefore, in this study, we turned to online recruitment websites and performed a comprehensive analysis of the labor market for big data talent by analyzing job recruitment advertisements.

We apply labor market segmentation theories to the context of big data labor market, specifically focusing on two fundamental aspects: labor allocation and salary determination. Firstly, we identify three dimensions of big data labor market segmentation: individual sociodemographic, job-related and organizational characteristics. Through this segmentation, we aim to examine the distribution of big data talent across various labor market segments, thereby addressing the following research questions regarding the Chinese big data labor market:

RQ1: Which industries and enterprises are actively recruiting big data talent? What types of jobs have a great need for big data? What types of big data talent are the most popular?

Secondly, we apply a salary determination model to the big data labor market, aiming to reveal salary variations across different segments of the big data talent market. This insight will enable us to identify the key factors influencing the salaries of big data talents in diverse market segments. Consequently, we seek to explore the following research questions:

RQ2: Are there significant differences in terms of salaries among big data talent in different labor market segments? Which factors have a significant impact on their salaries?

## Literature review

### Big data jobs

The term big data was first proposed by John Mashey in 1998 and has evolved through three distinct phases since the 1990s [16]. Phase 1.0 originated from traditional database management, emphasizing data storage, extraction, and optimization techniques within relational database management systems (RDBMS). Phase 2.0, emerging in the early 2000s with the expansion of web traffic, introduces challenges in handling semi-structured and unstructured data, especially from social media sources. Phase 3.0, the current era, emphasizes the significance of mobile devices and the Internet of Things (IoT), generating vast amounts of behavioral, location-based, and sensor data. This phase presents new opportunities and challenges in extracting valuable information, particularly in fields like transportation, healthcare, and urban planning. Big data usually refers to data of enormous size that cannot be easily captured, managed and processed by common techniques or software within tolerable time limits [17]. A definition published in 2016 stated that big data represents the information assets characterized by such high volume, velocity and variety that they require specific technology and analytical methods to be transformed into value [18]. Kaplan and Haenlein defined the term big data as data sets characterized by large amounts (volume) of frequently updated (velocity) data in various formats, such as numeric, textual, or images/videos (variety) [19]. Big data has had a major impact on human life, and its importance is recognized by people all around the world in many fields, including science, economics, culture and society. Big data provides a major opportunity to utilize rapidly upgraded information technologies to create value in many fields, especially in business. For example, Davenport noted that big data could be used to help enterprises reduce costs, improve their products and services, and support decision-making [20]. Therefore, an increasing number of enterprises are showing strong interest in big data. Thus, the big data industry is expected to have strong business potential in the future [21].

In recent years, there have been an increasing number of enterprises that have recognized the value of big data [22] and have attempted to employ big data talent, resulting in rapid growth in the demand for big data talent. The study from IBM announced that the company was planning to cultivate millions of scientific research and technical professionals who can work on big data projects and application systems in the next decade [23]. Cao [24] noted that the era of data industrialization is coming. While data industrialization creates new business value, traditional forms of commerce such as retail and manufacturing are giving way to new businesses with big data as a key driver.

A big data job refers to a position or role that typically require working with datasets characterized by the three Vs: volume (the amount of data), velocity (the speed of data processing), and variety (the number of types of data). These positions involve managing and analyzing vast amounts of data that are not only immense in quantity but also diverse in type and format, and generated at a high speed. These datasets often exceed the capabilities of traditional data-processing systems due to their scale and complexity, requiring specialized skills and tools for effective handling and analysis [25]. Big data roles can include data scientists, data engineers, or big data analysts, who are responsible for managing, processing, and analyzing vast amounts of data using various tools, technologies, and analytical techniques. They may also develop algorithms and models to analyze the data and identify patterns, trends, and relationships that can be used to make predictions and inform business decisions. Mauro et al. [26] built a big data job family and suggested that big data jobs can be broadly categorized into two separate groups: Technology-Enabler professionals and Business-Impacting professionals. The former group focuses on big data techniques, designed to extract economic value from vast and diverse datasets by enabling high-velocity capture, discovery, and analysis. These jobs include massively parallel processing, data mining tools and techniques, distributed file systems and databases, cloud computing platforms and scalable storage systems [27, 28]. The latter group focuses on big data analytics and applications, which can help organizations better understand the information contained within the data and make better and faster decisions by using advanced analytics techniques such as text analytics, machine learning, predictive analytics and data mining [29].

The existing literature primarily focus on the supply side of big data roles, emphasizing the technical aspects and skill sets required for managing and analyzing large and complex datasets. However, there is a notable absence of discussions regarding the demand side of the big data jobs, such as identifying industries and enterprises actively recruiting big data talent, the types of jobs with a high demand for such talent, and the most popular skill sets within the big data domain. This gap in analysis limits a comprehensive understanding of the dynamics and requirements of the big data job market. Addressing these aspects is crucial for aligning educational programs, skill development initiatives, and career strategies with industry needs, thereby fostering a more efficient and effective utilization of big data talent. Therefore, further research is needed to comprehensively analyze the demand side of big data jobs, providing a holistic understanding of the job market and informing talent acquisition and workforce development strategies.

### Labor market theory and models

The rapid development of the big data industry suggests that more attention should be devoted to the construction of a theoretical framework for understanding the big data labor market. Some theories of labor market segmentation have been proposed in the literature to explore the causes of labor market segmentation and how important those factors are to the distribution of wages [30]. Classical labor market theory assumes that individual workers can freely choose from among a wide range of job options in the labor market based upon their personal tastes, preferences, abilities, and skills [31]. It has been suggested that labor markets are segmented due to individual sociodemographic factors, including age, gender, race, and educational background, which cause labor inputs to be imperfect substitutes for each other [32]. However, subsequent studies have shifted the emphasis away from the supply side of the labor market and placed the focus on the demand side [33]. Scholars have realized that the labor market is not a single competitive market but is composed of a variety of noncompeting segments between which organizational or job-related barriers prohibit the population from benefiting equally from education and training [34]. These new segmentation theories argue that labor markets are segmented not only due to mere skill, experience and competence differentials, as these define only the heterogeneous nature of labor as a factor of production, but also due to the restricted movement of labor between and within industries [35]. Researchers, therefore, have resorted to using a number of different criteria to define labor market segments by considering organizational and job-related factors more than individual sociodemographic characteristics as the main causes of segmentation [36]. Some researchers have used organizational characteristics to define segments [37]. They have emphasized the importance of organizational influences on employment [38]. Internal labor market theory argues that the labor market is not a single competitive market. Some firms with internal labor market structures contain only good jobs that are characterized by high wages, good working conditions, stable employment, equity and opportunities for advancement. Jobs in other firms, in contrast, tend to have low wages and fringe benefits, poor working conditions, high labor turnover and few opportunities for advancement. Some researchers have used job-related characteristics to define segments. Occupational segmentation is widely considered to be an important dimension of labor market segmentation [39, 40]. Occupations are divided in many studies into different segments by setting subjective thresholds for levels of General Educational Development (GED) and Specific Vocational Preparation (SVP) corresponding to jobs listed in the Dictionary of Occupational Titles (DOT).

The main focus of labor market segmentation theory is wage differentials across labor markets. Much of the labor market literature is devoted to exploring the causes of market segmentation and how important those factors are for the distribution of wages [41]. Neoclassical theory assumes that individual workers receive rewards on the basis of their human capital endowments [31]. This theory emphasizes worker heterogeneity, including differential investments in human capital, rather than differences among jobs, as the primary cause of wage differentials. What this implies is that one worker may earn more than another in the labor market because he is more highly educated or skilled. In other words, low-wage jobs are filled by low productivity workers who are unable or unwilling to obtain the necessary skills that would allow them to access more highly paid jobs. However, in human capital theory, the determinants of wages have focused primarily on factors affecting labor supply. Demand factors have been relatively neglected. Some later segmentation literature has examined demand-side influences on the earnings of individual workers [42]. This research argues that rewards to human capital differ because organizational barriers prohibit the population from benefiting equally from education and training. Many researchers have attempted to test the hypothesis that wage determination mechanisms are different among labor market segments on the basis of job or industry characteristics [43]. The basic idea in these studies is to construct a wage differentials model to study how the factors in the different dimensions of labor market segmentation affect the determination of wages. Regression analysis is usually employed to examine differences in the wage determination process for each segment. Some favorable results to the segmentation hypothesis have recently been obtained that show that both supply-side and demand-side factors are important determinants of wage differentials [44].

The existing literature demonstrates that the labor market segmentation theory has been extensively utilized to identify distinct segments of the labor market based on various factors such as occupation, geography, skills, gender, race, education, and industry-specific characteristics. However, the application of this theory to the big data job market remains relatively unexplored. Given the dynamic and diverse nature of big data roles across industries, organizational scales, and skill requirements, there is significant potential for applying labor market segmentation theory to gain insights into the complexities and inequalities within this specific segment. By applying this theory to the big data job market, researchers and practitioners can gain profounder understandings of how factors like skills, qualifications, organizational sizes, geographical regions, and employment arrangements contribute to variations in demand and salaries among big data professionals.

### Content analysis of job advertisements

Many studies have attempted to understand the knowledge, skill and ability requirements of big data jobs by analyzing candidate requirements written in job advertisements. Some earlier studies mainly focused on traditional media advertisements. For example, Todd et al. [45] analyzed job advertisements placed in four major newspapers to examine changes in the knowledge and skill requirements of information system positions. They conducted statistical analyses of the frequency of phrases used in these advertisements to reach conclusions about the evolution of IS job skills. Similar studies of the job market have been conducted based on newspaper advertisements [46, 47].

With the rise of online recruitment websites such as Monster.com and Hotjobs.com, later studies have expanded their information sources from traditional media advertisements to online advertisements. Gallivan et al. [48] collected job advertisements from multiple sources, including newspapers, journals and the online job website Monster.com, to analyze changing patterns in IT skills. Wade and Parent [49] collected job descriptions from five online sites to identify the technical and organizational skills required by employers. In recent years, some researchers have attempted to use text-mining techniques to automatically extract key skill terms from the huge number of online job advertisements. For example, Debortoli et al. [50] applied text mining to analyze job advertisements from the website Monster.com and developed a skill taxonomy for big data. Fionn and Keith [51] focused on the website DataScientistJobs, which is a British recruiting website mainly for big data talent, and they sent email advertisements that recommended appropriate big data jobs to over 9,000 recipients every week. Their study analyzed job advertisements and identified 21 types of software skills that are the main concerns of employers.

The existing research on the context analysis of big data job advertisements has predominantly focused on extracting technical skills and abilities required for such roles, neglecting other valuable information present in job advertisements, including job location, responsibilities, education and work experience requirements. This narrow focus limits our comprehensive understanding of the actual market demand for big data talents. Moreover, the salary and compensation packages specified in these advertisements, which are essential for understanding compensation levels in the big data talent market, receive inadequate attention. Our research aims to bridge this gap by conducting a comprehensive analysis that considers not only technical skills but also incorporates these additional dimensions of job requirements and rewards. This approach not only enhances our understanding of the actual market demand for big data talents but also provides valuable insights for organizations in attracting and compensating big data talents effectively.

## Materials and methods

### Research design

This study applies labor market segmentation theories to analyze the labor market and salary determinants for big data talent in China. The widespread application of big data across industries demands a diverse range of knowledge and skills, rendering traditional methods inadequate for understanding the job market comprehensively. Labor market segmentation theories provide a framework to analyze these complexities, enabling a deeper understanding of various segments within the big data labor market and the factors influencing talent distribution and salaries. Therefore, we develop a theoretical framework based on labor market segmentation theory, particularly focusing on labor allocation and wage determination, to gain insights into the distribution of big data talent across different labor market segments and identify the key factors influencing their salaries. This is done in two steps.

First, the existing literature suggests that there are important differences in the criteria and methodology used to define and create segments in the labor market [52]. In fact, methods based solely on any single dimension of the market, such as occupation, industry, firm, or job characteristics, cannot adequately capture the essence of labor market segmentation. Based on the literature reviewed, we identify three dimensions of big data labor market segmentation: individual sociodemographic, job-related and organizational characteristics.

Second, we apply the wage differentials model to the context of the big data labor market to examine the determinants of the salaries of big data talent. Considering that wage differential models based on different segmentation methods might produce different results, we construct a regression model of wage determination that attempts to explain wage differentials on the basis of a wide range of individual, job-related and organizational characteristics.

Two individual characteristics are included in the regression analysis: education and work experience. Human capital theory suggests that a large proportion of the variation in earnings can be explained by differences in education, training, and work experience [53]. It is assumed that better educated workers are also more productive and that educational level is a reliable indicator or signal of higher productivity [54]. Work experience is used as a reasonable proxy for non-observable investments in on-the-job training. Employers are willing to pay a higher salary to those with rich work experience and a good educational background to reduce training costs and improve productivity [55]. Therefore, we propose the following hypothesis:

H1: Individual characteristics, including level of education and work experience, have a significant influence on wage differentials in the big data labor market.

Two job-related characteristics are included in the regression analysis: occupation and job level. Employers are willing to pay a higher salary to those employees in skill-intensive occupations, while they tend to pay low wages to workers in labor-intensive occupations to avoid the sunk costs of capital investment and labor training [29]. Higher wages are also often paid to workers in occupations with unattractive job attributes [56], such as monotony or a high probability of accident occurrence, because dangerous or risky working conditions necessitate the payment of a compensating differential to workers [57]. In addition, job level is recognized in many wage determination models as an important determinant of wage differentials. Therefore, we propose the following hypothesis:

H2: Job-related characteristics, including occupation and job level, have a significant influence on wage differentials in the big data labor market.

Three organizational characteristics are included in the regression analysis: location, industry, and ownership. The existence of regional wage differentials [58], interindustry wage differentials [59] and public-private sector wage differentials [60] has been well documented in the literature. Regional wage differentials might compensate for differences in the cost of living and urban amenities across regions [61]. Interindustry wage differentials can be explained by noncompetitive labor market theories and efficiency wage theories [62]. Public-private sector wage differentials can be explained by the different natures of the institutions that regulate wage determination in the private and public sectors [63]. Private employers are profit-maximizing agents, while public employers have different goals when making labor demand decisions. Some factors may lead to lower public sector wages, but nonwage advantages, such as generous pension plans, may compensate for lower wages in the public sector [64]. Therefore, we propose the following hypothesis:

H3: Organizational characteristics, including location, industry, and ownership, have a significant influence on wage differentials in the big data labor market.

### Data collection

The data in this study were retrieved from Zhaopin.com, one of the leading online recruitment websites in China. The market coverage for Zhaopin.com is now approximately 28 percent of the Chinese online recruitment market. The site meets the recruitment needs of users in different industries. Furthermore, the site emphasizes that they serve job seekers in more than 60 industries and provide jobs for approximately 600 subdivided occupations in nearly 40 job categories. The website provides professional HR services to over 2.2 million clients, and its average daily page views are over 68 million. In the past 12 months, more than 40 million job advertisements have been placed on the website by multinational corporations, small and medium-sized enterprises and state-owned enterprises. Job advertisements on the website usually contain the following information: job title, company name and introduction, job description, candidate requirements and some additional information including workplace, salary, bonus opportunities, and benefits.

**Fig 1:**
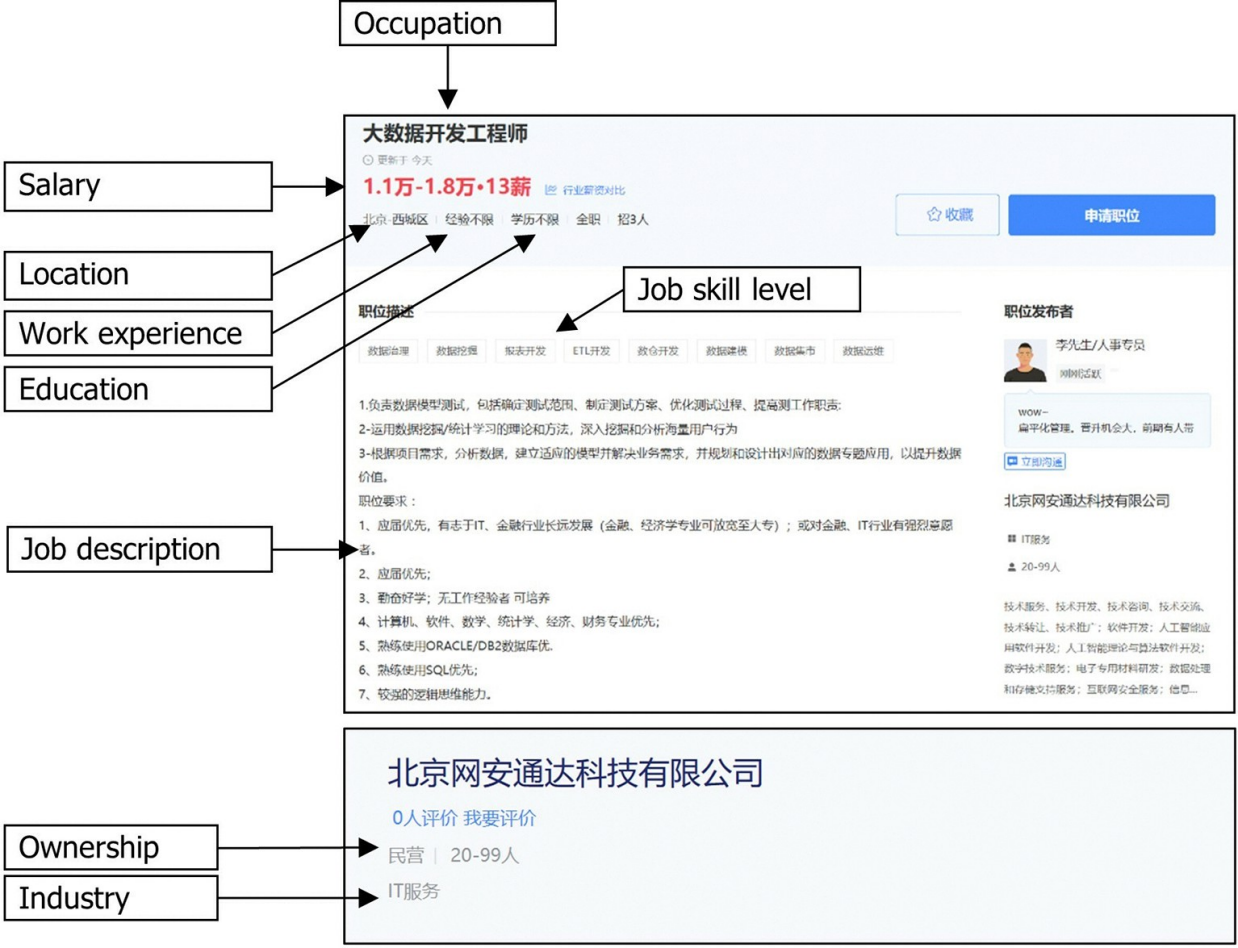
A screenshot of a big data job advertisement page on the site.

We collected useful information from Zhaopin.com and then stored that information in our database as sample data. The procedure was as follows: First, we used the keyword “big data” as a query to search for all job advertisements related to big data. Fig 1 shows a screenshot of a big data job advertisement page when searching for “big data” on the site. The site provides only the results list of job advertisements where the term “big data” occurs in the job title or is used as a keyword in the position description. Therefore, the retrieved job advertisements are all closely related to big data. Next, we downloaded all the webpages where job advertisements were posted from March 1 to March 31 in 2023 by using a self-developed web crawler. Then, we parsed the files to extract the available metadata from job advertisements and stored them in a database. As indicated in Fig 1, we extracted the following data from each job advertisement: occupation, job skill level, salary, location, work experience, education, ownership, and industry. Table 1 provides the description of available data in a job advertisement. Thus, we obtained 13,392 job records in total. Then, to improve the quality of the data, we preprocessed the raw records. After removing 2,132 duplicate job records and 3,504 records with missing values, a total of 7,756 job recruitment records were selected as the final sample.

**Table 1:** Description of the available data in a job advertisement.

Variable	Description
Education	The academic credentials or degrees the individual must have obtained
Work experience	The number of years that individuals have been engaged in big data jobs
Occupation	The type of big data-related job positions
Level	The skill level required for the job
Location	The city where the organization is located
Industry	The industrial category of the organization
Ownership	The ownership type of the organization
Salary	The monthly salary measured in tens of thousands of Chinese yuan

### Empirical approach

In this paper, an empirical analysis is performed on online job advertisements related to big data in order to investigate the labor market for big data talent in China. In our work, we first analyze the different dimensions of big data labor market segmentation and then perform a descriptive statistical analysis to investigate the distribution of big data talent. Finally, multiple regression analysis is employed to investigate which factors have a significant impact on salaries for big data jobs. We identify three dimensions of big data labor market segmentation: individual sociodemographic, job-related and organizational characteristics.

The individual characteristics used in our research include education and work experience. (i) Education. Education refers to the academic credentials or degrees an individual has obtained. Education levels for talent are categorized into five classes: high school & lower, associate’s degree, bachelor’s degree, master’s degree and Ph.D. It is well known that highly educated individuals are better qualified for big data jobs because these jobs usually require higher levels of professional knowledge and skills. A survey conducted by O’Reilly Media, a leading computer and technical book publisher, found that studying for a Ph.D. and mastering additional analytical tools were the most effective ways for data analysts to increase their salaries. It was estimated that individuals could expect to increase their salaries by 1900 US dollars per year when they master one more new big data analysis tool [65]. (ii) Work experience. Work experience in our study refers to the total number of years that the individual has been engaged in big data jobs. It is obvious that experienced employees are more likely to be desired by employers and to obtain higher salaries, especially in big data-related positions requiring strong professional skills.

The job-related characteristics used in our research include occupation and job level. (i) Occupation. There are many types of occupations that require big data talent, e.g., big data system development and maintenance, big data application development and big data analysis. The demand for big data talent in different types of job positions is not distributed equally. We choose the PRC Grand Classification of Occupations (CGCO) as the occupational classification standard. The new CGCO was released jointly by the Ministry of Human Resources and Social Security (MOHRSS), the State Administration for Market Regulation (SAMR), and the National Bureau of Statistics (NBS) in 2015. The classification system contains 8 major categories, 434 subcategories, and 1,481 occupations, which is used to provide guidance for occupational education and job qualification criteria for all industries. The CGCO is officially defined and is often used in many studies as the occupational classification standard for job market analysis. (ii) Job skill level. Big data talent at all job levels is desperately needed because the supply of talent cannot keep up with demand, especially in high-level jobs. It will take significant time and effort to train people for high-level job tasks, resulting in a serious shortage of high-level big data talent. Meanwhile, the rapid growth of the big data industry is creating a substantial increase in demand for low-level big data talent. According to these regulations, professional and technical positions in China are divided into three grades, including junior, intermediate, and senior positions. Thus, we apply the same principles to divide big data-related job positions into the same three levels.

The organizational characteristics used in our research include location, industry, and ownership. (i) Location. The National Bureau of Statistics (NBS) of China focuses on 70 large and medium-sized Chinese cities and categorizes these cities into three tiers, mainly based on key characteristics of the cities such as city size, population, and GDP. According to the NBS, China has four first-tier cities: Beijing, Shanghai, Guangzhou, and Shenzhen. Capital cities of provincial regions and some other major cities are listed as second-tier cities, including Hangzhou, Chengdu, and Xiamen. There are a total of 31 cities listed as second-tier cities. The other 35 cities are listed as third-tier cities. The division of Chinese city grades by NBS has been widely recognized, so we also use these same divisions to classify the cities into first-, second- and third-tier cities. (ii) Industry. The current official industry classification system in China is GB/T 4754–2017, which is the Industrial Classification for National Economic Activities in China and which follows the International Standard Industrial Classification of All Economic Activities (ISIC) Revision 4. The classification system was published by the National Bureau of Statistics (NBS) in China in 2017. According to GB/T 4754–2017, economic activity in China is divided into three industries: the primary, secondary, and tertiary industries. The primary industry consists of agriculture, forestry, animal husbandry, fisheries, and so on. The secondary industry includes mining, manufacturing, construction, and so on. The tertiary industry is also named the service industry, including entertainment, education, legal, medical, and financial services. We use this system to classify industries into these three categories. (iii) Ownership. Different types of organizations, including private enterprises, state-owned enterprises, listed enterprises and other enterprises, have different demands for big data talent.

## Results

### Descriptive statistics for big data jobs

Table 2 provides the overall descriptive statistics for our sample. The demand for big data talent is highest for those with a bachelor’s degree, accounting for 65.32%, and for those with 3–5 years of work experience, accounting for 36.78%. The proportion of demand for big data talent from second-tier cities is 60.42%, followed by that for first-tier cities with a proportion of 37.83%, both of which are far greater than that of the third-tier cities. This finding shows that there is a serious imbalance in the distribution of demand for big data talent in different cities in China and that first- and second-tier cities have a greater demand for big data talent. Only 34 job advertisements are from the primary industry, accounting for 0.44% of all advertisements, and 1,912 job advertisements are from the secondary industry, accounting for 24.65%. In contrast, enterprises in the tertiary industry published the largest number of recruitment advertisements, accounting for 74.91%. This indicates that the tertiary industry has the greatest demand for big data talent.

**Table 2:** The overall descriptive statistics for the sample.

	Count	Percentage (%)
Education:		
No requirements	164	2.11
< = High School	37	0.48
Associate’s degree	2,202	28.39
Bachelor’s degree	5,066	65.32
Master’s degree	259	3.34
Ph.D.	28	0.36
Experience:		
< = 1	1,694	21.84
1–3	2,054	26.48
3–5	2,835	36.78
5–10	1,155	14.89
Occupation:		
Technology & Development	4,582	59.08
Operations & Management	705	9.09
Design & Analysis	2,433	31.37
Marketing & Service& Other	36	0.46
Level:		
Junior	2,785	35.91
Intermediate	2,518	32.47
Senior	2,453	31.63
Location:		
First-tier	2,934	37.83
Second-tier	4,686	60.42
Third-tier	136	1.75
Industry:		
Primary	34	0.44
Secondary	1,912	24.65
Tertiary	5,810	74.91
Ownership:		
Private	4,570	58.92
State-owned	732	9.44
Listed	1,316	16.97
Other	1,138	14.68

**Fig 2:**
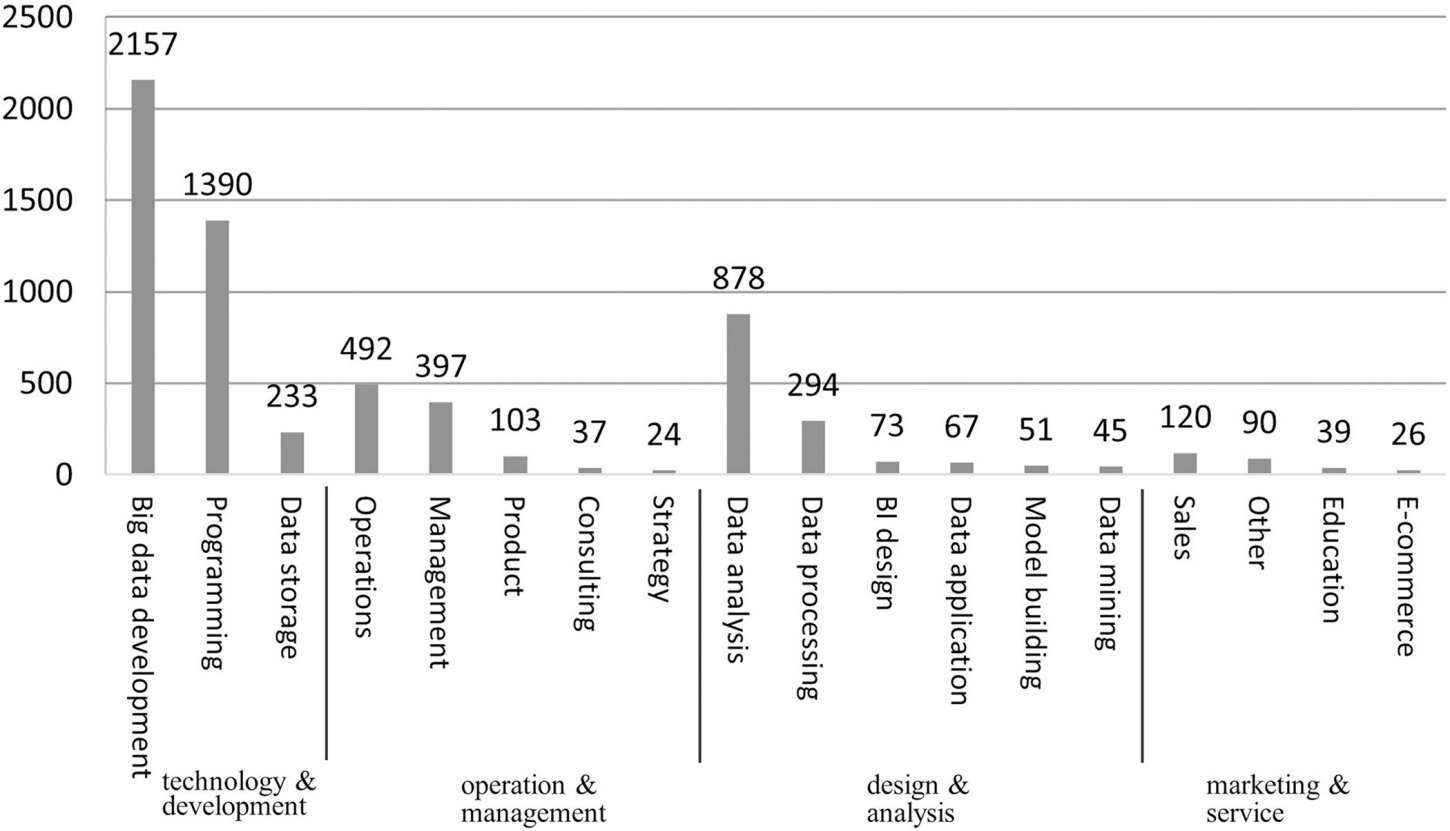
Sample sizes for 18 types of big data jobs positions.

Using the job titles and classification system provided by the site, we found that the job advertisements in our sample could be classified into 3,444 types of job positions closely related to big data. These job positions were further classified into 18 types of jobs. The sample size of each type of job is shown in Fig 2. According to the occupational categories from the Chinese Grand Classification of Occupations (CGCO), we further aggregated the 18 types of jobs into four occupations: technology & development, operations & management, design & analysis, and marketing & services. As indicated in Fig 2, technology & development positions account for the largest proportion of all positions, while the other three categories account for only a small proportion of all positions. Especially in the marketing & services category, there is less demand for big data jobs. On the whole, there seems to be a growing demand for diversification of big data talent in the Chinese job market.

**Fig 3:**
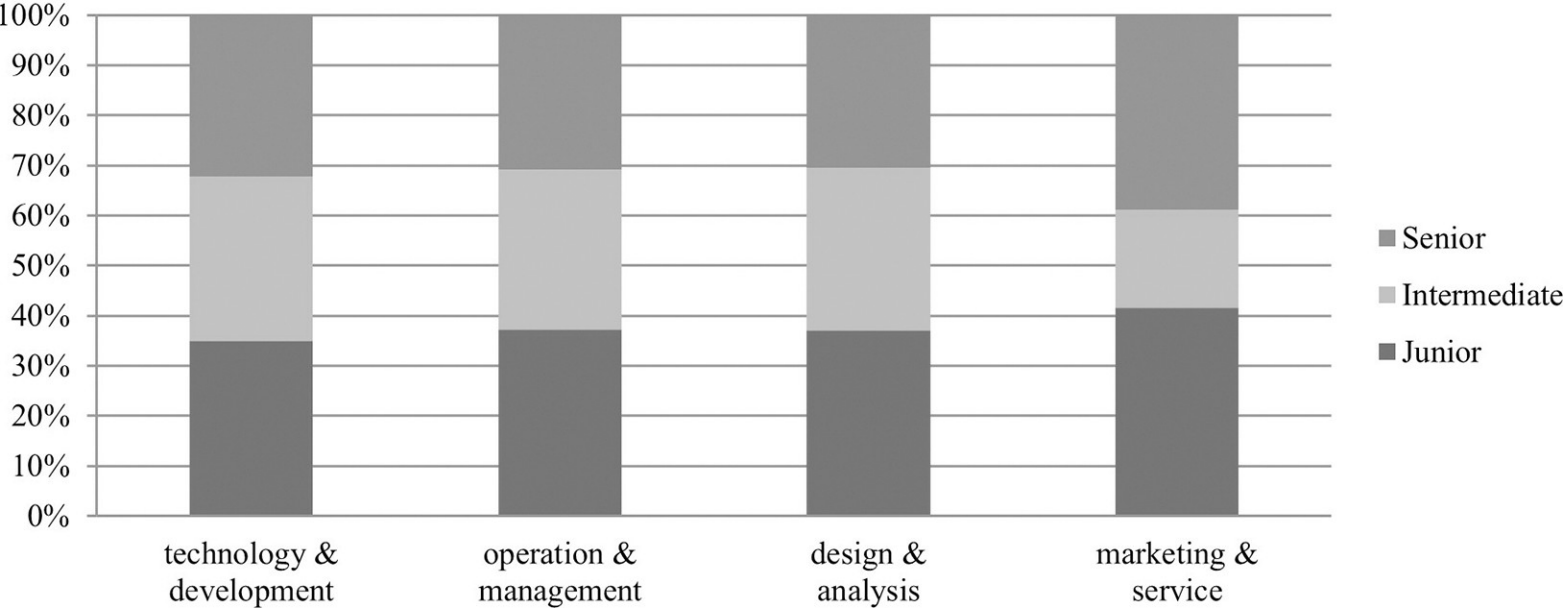
Proportion of junior, intermediate, and senior positions across four categories of big data jobs.

Further analysis was performed to investigate the distribution of job levels in different big data job categories. Big data-related job positions can be classified into three levels: junior, intermediate and senior positions. Because no subject-specific or national general standards exist, the position level of a given big data-related job needs to be inferred from the job skill level. For junior job positions, the required skills typically include business negotiation, sales, solution design, human resources, data extraction, finance, and so on. For intermediate job positions, common skill requirements include Java, Spark, Hadoop, digital warehousing, cloud computing, and data modeling. For senior job positions, the required skills typically include data crawling, data mining, big data development, financial data analysis, business data analysis, data governance, and data operations and maintenance. This study calculates the total score of skill difficulty based on the job skill descriptions and then classify positions into different levels based on the scores. The result is shown in Fig 3. There are significant variations in the distribution of job levels among the four categories of big data jobs. The proportion of junior job positions is the lowest in the technology & development category, but the highest in the marketing & service category. The proportion of intermediate job positions is significantly lower in the marketing & service category than in the other categories, while the proportion of senior job positions is notably higher in the marketing & service category than in the other categories. In the operation & management and design & analysis categories, the proportions of junior, intermediate, and senior job positions are relatively similar.

**Fig 4:**
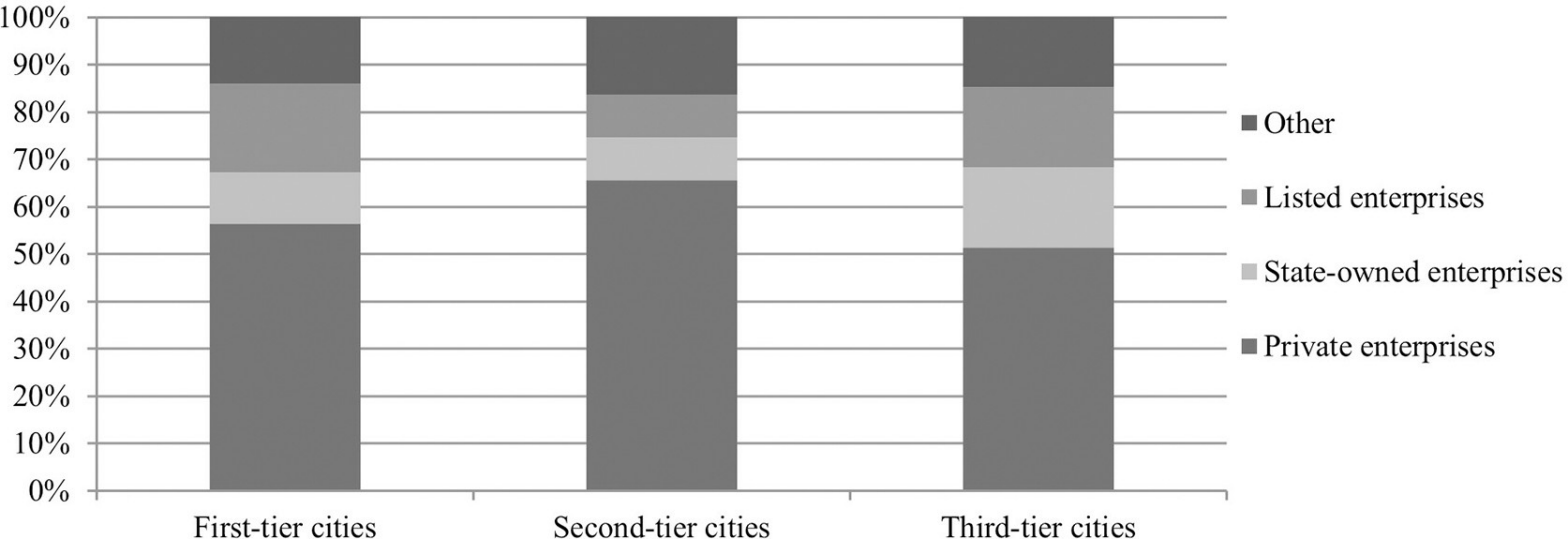
Proportions of big data talents across four types of organizations in first-, second-, and third-tier cities.

The distribution of organizational ownership types in different city grades is shown in Fig 4. We can see private, state-owned, and listed enterprises have different demand patterns for big data talents across different tier cities. Private enterprises tend to have a higher demand in second-tier cities, state-owned enterprises tend to have a higher demand in third-tier cities, and listed enterprises tend to have a higher demand in first-tier cities.

### Empirical analysis of factors affecting the salaries of big data talent

We developed an empirical model with salary as the dependent variable and employ multiple regression analysis to investigate which factors have a significant impact on salaries for big data jobs. As the dependent variable in our empirical analysis, salary was measured as a continuous variable using monthly salary. The independent variables in our empirical model are the factors affecting salary, which have been discussed in the Research design section.

The variable education was measured using a five-level (1 through 5) ordinal scale (1 = high school or below, 2 = associate’s degree, 3 = bachelor’s degree, 4 = master’s degree, and 5 = Ph.D.). We used the average educational level of available samples to estimate the missing education requirement data in job recruitment records that do not have specific educational requirements. The variable work experience was measured as a continuous variable representing the number of years that the individual has been engaged in big data jobs. The variable occupation was classified into four categories: technology & development, operations & management, design & analysis, market & service. We thus used four binary variables with 1 for yes and 0 otherwise to measure the different types of jobs. The variable job level was measured using a three-level ordinal scale (1 = junior, 2 = intermediate, and 3 = senior). The variable location is measured using a three-level (1 through 3) ordinal scale (1 = third-tier cities, 2 = second-tier cities, and 3 = first-tier cities). The variable industry was measured using a three-level ordinal scale (1 = primary industry, 2 = secondary industry, and 3 = tertiary industry). The variable ownership was classified into four categories: private enterprises, state-owned enterprise, listed enterprises, and others. We thus used four binary variables with 1 for yes and 0 otherwise to measure the different types of organizational ownership.

Table 3 presents the correlation matrix for all the measured variables, including the dependent variable and the independent variables. As shown in Table 3, there is a high, positive correlation among work experience, educational background, job level, and location, indicating that talent who have rich work experience and higher levels of education are more highly demanded in economically developed cities and are more likely to obtain senior job positions.

**Table 3:** Correlation matrix for all variables.

	ED	WE	TD	OM	DA	MS	JL	LO	IN	PE	SE	LE	OE	SA
ED	1													
WE	.204**	1												
TD	.032**	.027*	1											
OM	-.026*	-.030**	-.380**	1										
DA	-.02	-.011	-.812**	-.214**	1									
MS	.013	.009	-.082**	-.022	-.046**	1								
JL	.075**	.134**	.022	-.008	-.018	.001	1							
LO	.047**	.096**	.125**	-.051**	-.098**	-.018	.064**	1						
IN	.006	.036**	.021	-.019	-.008	-.016	.087**	.059**	1					
PE	-.223**	-.121**	-.012	.017	.004	-.009	.022	-.033**	.005	1				
SE	.155**	.063**	.011	-.015	-.002	-001	-.042**	.024*	-.003	-.387**	1			
LE	.135**	.123**	.017	-.016	-.01	.01	.016	.039**	-.009	-.541**	-.146**	1		
OE	.039**	-.013	-.01	.006	.006	.01	-.013	-.015	.005	-.497**	-.134**	-.187**	1	
SA	.068**	.087**	.321**	-.142**	-.247**	-.034**	.071**	.273**	.050**	-.032**	.029**	.056**	-.038**	1

Note

* indicates a significant correlation at the 0.05 level

ED = education, WE = work experience, TD = technology & development jobs, OM = operations & management jobs, DA = design & analysis jobs, MS = marketing & service jobs, JL = job level, LO = location, IN = industry, PE = private enterprises, SE = state-owned enterprises, LE = listed enterprises, OE = other enterprises, and SA = salary.

Based on the correlation between occupation and other variables, it can be inferred that technology & development job positions require more work experience and higher education while operations & management job positions have relatively low requirements for work experience and education. Moreover, technology & development jobs tend to be concentrated in higher-tier cities, while other types of positions are more prevalent in lower-tier cities.

From the correlation between organizational ownership types and other variables, we can conclude that state-owned and listed enterprises are more likely to look for big data talent with rich work experience and better education. In contrast, private enterprises have relatively low requirements in terms of work experience and education. Additionally, location displays a significant positive correlation with state-owned and listed enterprises, while a significant negative correlation with private enterprises. This implies that the demand for big data talent from state-owned and listed enterprises is mainly centered in economically advanced cities such as first- and second-tier cities. Conversely, the demand for big data talent from private enterprises is primarily focused on economically underdeveloped cities like third-tier cities.

We emphasize the correlation between salary and the independent variables to determine the impact of various factors on salary. As shown in Table 3, there is a high, positive correlation between work experience, educational background, job level, location, and salary. These results show that big data talent with more work experience and a higher level of education is more likely to obtain a higher salary. Meanwhile, working in higher-level job positions or in economically developed cities will help individuals increase their salaries. In addition, based on the correlation between salary and four types of job positions, we can infer that big data talents working in technology & development positions are likely to earn higher salaries than the average, whereas those in other types of positions may receive lower salaries than the average. In the same way, based on the correlation between salary and the four types of organizational ownership, we can infer that salaries in state-owned and listed enterprises are generally higher than those in private enterprises and other types of enterprises.

Table 4 presents the results of the multiple regression analysis of salary. It should be noted that because there are four binary variables used to measure organizational ownership (private enterprises, state-owned enterprises, listed enterprises and other enterprises), we could only add three variables, private enterprises, state-owned enterprises and listed enterprises into the model, while job advertisements from other enterprises served as the control group to avoid multicollinearity. In the same way, because there are four binary variables used to measure occupation, we could only add three variables into the model, while marketing & service category served as the control group to avoid multicollinearity.

**Table 4:** Multiple regression analysis for salary.

	Variables	Coefficient	Std. error	T	Sig
Individual	Education	270.85**	94.02	2.88	0.004
	Work experience	78.20**	21.19	3.69	0.000
Job-related	Occupation				
	Design & Analysis	546.72	713.49	0.77	0.444
	Operations & Management	205.66	726.15	0.28	0.777
	Technology & development	3191.39**	711.24	4.49	0.000
	Job level	227.07**	59.790	3.80	0.000
Organizational	Location	2034.72**	95.05	-21.41	0.000
	Industry	264.78*	108.79	2.43	0.015
	Ownership				
	Private enterprises	318.44*	141.48	2.25	0.024
	State-owned enterprises	571.65**	202.54	2.82	0.005
	Listed companies	690.35**	172.85	3.99	0.000
Constant		13005.36**	842.33	15.44	0.000
Number of observations		7,756			
R-squared		0.167			
Adjusted R-squared		0.166			
F-value		141.38**			

Notes

** p<0.01

* p<0.05

From Table 4, we can see that the F-value of 141.38 is significant (p<0.01), indicating that the overall fit of the regression model is statistically significant at this level. The t-test is used to test whether the independent variables have a significant effect on the dependent variable. As indicated in Table 4, the independent variables are all significant predictors of salary. The detailed analysis is as follows.

First, we sought to investigate whether individual characteristics have a significant impact on salaries for big data jobs. The results show that the coefficient on education is positively associated with salary (B = 270.85, p<0.01), indicating that big data talent with a higher education level is more likely to obtain a higher salary than talent with a lower education level and that the increase in salary is 270.85 yuan. In addition, the results show that the coefficient on work experience is positively associated with salary (B = 78.20, p<0.01), indicating that big data talent with more work experience obtains a higher salary than talent with less work experience, and such workers can earn an extra 78.20 yuan per month with an increase in their working years. Thus, hypothesis H1 is supported, and we conclude that individual characteristics, including level of education and work experience, have a significant influence on wage differentials in the big data labor market.

Next, we sought to investigate whether job-related characteristics have a significant impact on salaries for big data jobs. We use marketing & service category as the control group in order to compare the salaries for marketing & service jobs with those for the other three types of jobs. The results show that the coefficients on technology & development are positively associated with salary (B = 3191.39, p<0.01 for technology & development), indicating that the salaries in technology & development jobs are 3191.39 yuan higher than in marketing & service jobs. We further examine whether job level can have a significant impact on salary and found that big data talent in higher-level job positions is more likely to obtain a higher salary than that in lower-level job positions, and the increase in salary is 227.07 yuan according to the results showing that the coefficient on job level is positively associated with salary (B = 227.07, p<0.01). Thus, hypothesis H2 is supported, and we can conclude that job-related characteristics, including occupation and job level, have a significant influence on wage differentials in the big data labor market.

Finally, we sought to investigate whether organizational characteristics have a significant impact on salaries for big data jobs. The results show that the coefficient on location is positively associated with salary (B = 2034.72, p<0.01), indicating that big data talent in higher-tier cities is more likely to obtain a higher salary than that in lower-tier cities and that the increase in salary is 2034.72 yuan. The coefficient on industry is positively associated with salary (B = 264.78, p<0.05), indicating that big data talent in the tertiary industry obtains the highest salaries, followed by those in the secondary industry and primary industry, and the decrease in salary is 264.78 yuan. A reasonable explanation is that big data is widely used in certain high-paying industries, such as IT and finance, which belong to the tertiary industry, so the salary in the tertiary industry is accordingly higher. For organizational ownership types, we used recruitment advertisements from other enterprises as a control group and found that the wages of private enterprises, state-owned enterprises, and listed enterprises were significantly higher than those of other enterprises. The results showed that the coefficient of wages between private enterprises, state-owned enterprises, and listed companies was positive (B = 318.44, p<0.05 for private enterprises; B = 571.65, p<0.01 for state-owned enterprises; B = 690.35, p<0.01 for listed enterprises). It is estimated that the average salary in private enterprises is 318.44 yuan higher than that in other enterprises and that the salary in state-owned enterprises is 571.65 yuan higher than that in other enterprises and that the salary in listed enterprises is 690.35 yuan higher than that in other enterprises. Thus, hypothesis H3 is supported, and we can conclude that organizational characteristics, including location, industry, and ownership, have a significant influence on wage differentials in the big data labor market.

## Discussion

We conducted an empirical analysis of online job advertisements on a Chinese recruitment website to investigate the distribution of big data talents across different labor market segments and identify the factors that significantly impact their salaries. Some valuable findings were obtained as follows.

First, we found that the rapid growth of the big data labor market and the high demand for big data talents across various industries. In contrast to many traditional high-paying jobs, which may have a more balanced supply-demand relationship, big data jobs have a significant imbalance in the supply and demand of big data talent, thereby resulting in a shortage of big data talents and increasing salaries. In addition, we observed a significant diversification in the demand distribution for different levels and skills of big data talents across different types of jobs positions. Contrary to other high-paying jobs, employers seeking big data talents in various industries require a broad range of skills, including data collection, analysis, and processing of big data, and may even require knowledge of specific business processes and domain expertise. Big data talents are expected to possess not only technical skills but also a deep understanding of the business context and domain knowledge to interpret and extract meaningful insights from large datasets. This combination of technical and domain knowledge makes big data jobs more diverse and complex than many other high-paying jobs.

Second, we investigated the distribution of big data talent market from several aspects, including individual, job-related, and organizational characteristics, and found that there is a significant distribution imbalance in the demand for big data talent market. Specifically, it is reflected in the following aspects: (1) There was a serious imbalance in the distribution of the demand for different levels of big data talent in different types of job positions. Technology & development jobs account for the largest proportion of all positions, followed by design & analysis positions, while the other two categories account for only a small proportion of all positions. The distribution of job levels varies significantly among the four categories of big data jobs. In the technology & development category, there are fewer junior job positions compared to the other categories. The marketing & service category has the highest proportion of junior and senior job positions and the lowest proportion of intermediate job positions. The operation & management and design & analysis categories have similar proportions of junior, intermediate, and senior positions. (2) There is a significant imbalance in the distribution of demand for big data talent based on the organizational characteristics of the big data labor market. For example, the demand for big data talent in the tertiary industry is much higher than that in the primary industry and secondary industry. First- and second-tier cities have greater demand for big data talent than do third-tier cities. Private enterprises tend to have a higher demand in second-tier cities, state-owned enterprises tend to have a higher demand in third-tier cities, and listed enterprises tend to have a higher demand in first-tier cities. (3) We investigated the individual characteristics of the big data labor market and obtained valuable conclusions. For example, talent with a rich work experience and higher-level education is more popular in economically developed cities and is more likely to obtain senior-level job positions. In addition, we conclude that state-owned and listed enterprises are more likely to look for big data talent with rich work experiences and better education. In contrast, private enterprises have relatively low requirements in terms of work experience and education.

Finally, we employed multiple regression analysis to investigate which factors can have a significant impact on salaries for big data jobs. Our empirical results reveal that individual, job-related and organizational characteristics are all significant predictors of salary. The results offer valuable insights as follows. Big data talent can increase earnings by working in higher-tier cities or in tertiary industries. Technology & development jobs offer higher salaries than other types of job positions. Big data talent with a higher level of education and more work experience is more likely to obtain a higher salary.

## Conclusions

### Theoretical and practical implications

This study has several significant theoretical implications for understanding the distribution of big data talent and the determination of their salaries. First, the study makes a valuable contribution to the existing literature on labor market segmentation by applying this theory to the context of big data talent. The theoretical framework developed in this study provides a new perspective for understanding the distribution of big data talent. Traditional labor market research typically focuses on the general supply and demand of labor, often neglecting the special features of different types of talents. In contrast, this study pays attention to the specific characteristics of big data talent, such as their high demand and special skills. Through empirical modeling, the study demonstrates that the distribution of big data talent is influenced by a variety of factors, including industry, regional demand, education, and so on. This perspective provides a more comprehensive understanding of the distribution of big data talent. Another theoretical implication is that the wage differentials of big data talent can be explained by a salary determination model that incorporates factors such as job requirements, skill levels, experience, industry demand, and regional economic development. This model supplements the traditional perspective that wages are primarily determined by labor market supply and demand forces, and emphasizes the importance of individual characteristics and job requirements in explaining wage differentials. We believe that this study will offer useful insights to labor market researchers, providing a basis for understanding how wages are determined in the context of a specific labor market, especially for the high-skilled talent market.

The insights gained from this research can contribute significantly to the advancement of the big data industry in China in several ways. First and foremost, understanding the distribution of big data talent across different labor market segments can help organizations devise more targeted recruitment strategies. For instance, identifying significant differences in the demand for big data talent across different types of cities and industries can help companies focus their recruitment efforts in those areas, thereby improving their chances of attracting the most suitable candidates. Secondly, recognizing that different types of enterprises have varying requirements for individual characteristics and offer various levels of big data job positions can aid in optimizing job positioning and role definitions within organizations. This can lead to more efficient utilization of human resources and better alignment between job roles and organizational needs. Thirdly, the findings on salary differentials based on individual, job-related, and organizational characteristics provide valuable insights for organizations in terms of compensation and reward strategies for big data talent. By understanding the factors that influence salaries, organizations can ensure that they offer attractive compensation packages to recruit and retain skilled professionals in the competitive big data market. Finally, the results of our study can serve as practical guidance for educational institutions and training providers. They can gain a deeper understanding of the high-demand jobs that require big data expertise and develop more suitable curricula and training programs to cultivate big data talent skills that align with the needs of the big data industry.

From a policy perspective, the government can leverage the findings of this research to formulate targeted policies that support the development of the big data industry. For instance, policies that encourage talent flow between cities or industries with high and low concentrations of big data talent can help balance the distribution of skills and expertise. policies that promote skill development and training in areas identified as critical for the big data industry can enhance the overall competitiveness of the sector. Policies that encourage collaboration between industry, academia, and research institutions can also help to foster a vibrant ecosystem for big data talent. Additionally, some further studies involve exploring the specific factors influencing the demand for big data talent in various cities and industries, as well as conducting longitudinal studies to track changes in the demand for big data talent over time. These efforts would provide valuable insights into the evolving dynamics of the industry and the effects of emerging technologies on job roles and skill requirements, facilitating policymakers in designing targeted interventions such as skill development programs, incentives for industry-academia collaborations, and policies promoting equitable access to job opportunities within the big data sector.

## Limitations and future work

This study has some limitations. First, this study only uses "big data" as the search term when searching and collecting big data job advertisement on the recruitment websites. This may result in overlooking some job postings closely related to big data that do not explicitly use the term "big data" in their descriptions. Additionally, there is a risk of including job advertisements that are not closely related to big data if they happen to contain the keyword. To address this limitation, future efforts may involve expanding the search terms or employing a semi-manual inspection process to further refine the sample, ensuring a more accurate representation of big data job postings. Second, this study identified some key factors affecting the salary of big data talents across personal, job-related, and organizational dimensions. However, there may be additional influencing factors, such as gender and specific skill requirements for the positions, that were not considered in this analysis. Future research should further extract valuable information on these factors by employing text analysis techniques on job descriptions and specifications to provide a more comprehensive understanding of the determinants of salary in the context of big data recruitment. Third, while this study provides a comprehensive analysis of the segmentation of the big data talent and the determinants of salaries, there is a need for future research to conduct comparative analyses with other high-salary industries that require similar high-level skills, particularly those closely related to big data such as data science and computer science. These comparative studies are essential for gaining a deeper understanding of the differences and similarities between big data labor markets and other high-skill, high-salary industries, facilitating the generalization of our conclusions.

## Supporting information

S1 Data (XLSX)
